# A straightforward and efficient analytical pipeline for metaproteome characterization

**DOI:** 10.1186/s40168-014-0049-2

**Published:** 2014-12-10

**Authors:** Alessandro Tanca, Antonio Palomba, Salvatore Pisanu, Massimo Deligios, Cristina Fraumene, Valeria Manghina, Daniela Pagnozzi, Maria Filippa Addis, Sergio Uzzau

**Affiliations:** Porto Conte Ricerche, S.P. 55 Porto Conte/Capo Caccia Km 8.400, Tramariglio 07041 Alghero, Italy; Department of Biomedical Sciences, University of Sassari, Viale San Pietro 43/B, 07100 Sassari, Italy

**Keywords:** Gut microbiota, Microbial community, Mouse, Metaproteomics, Protein extraction, Proteomic methods, Shotgun proteomics, Mass spectrometry, LC-MS/MS, Single-run liquid chromatography

## Abstract

**Background:**

The massive characterization of host-associated and environmental microbial communities has represented a real breakthrough in the life sciences in the last years. In this context, metaproteomics specifically enables the transition from assessing the genomic potential to actually measuring the functional expression of a microbiome. However, significant research efforts are still required to develop analysis pipelines optimized for metaproteome characterization.

**Results:**

This work presents an efficient analytical pipeline for shotgun metaproteomic analysis, combining bead-beating/freeze-thawing for protein extraction, filter-aided sample preparation for cleanup and digestion, and single-run liquid chromatography-tandem mass spectrometry for peptide separation and identification. The overall procedure is more time-effective and less labor-intensive when compared to state-of-the-art metaproteomic techniques. The pipeline was first evaluated using mock microbial mixtures containing different types of bacteria and yeasts, enabling the identification of up to over 15,000 non-redundant peptide sequences per run with a linear dynamic range from 10^4^ to 10^8^ colony-forming units. The pipeline was then applied to the mouse fecal metaproteome, leading to the overall identification of over 13,000 non-redundant microbial peptides with a false discovery rate of <1%, belonging to over 600 different microbial species and 250 functionally relevant protein families. An extensive mapping of the main microbial metabolic pathways actively functioning in the gut microbiome was also achieved.

**Conclusions:**

The analytical pipeline presented here may be successfully used for the in-depth and time-effective characterization of complex microbial communities, such as the gut microbiome, and represents a useful tool for the microbiome research community.

**Electronic supplementary material:**

The online version of this article (doi:10.1186/s40168-014-0049-2) contains supplementary material, which is available to authorized users.

## Background

Metaproteomics has been first defined by Wilmes and Bond as “the large-scale characterization of the entire protein complement of environmental microbiota at a given point in time” [1]. In less than a decade, in parallel with the remarkable progresses in mass spectrometry and proteome bioinformatics, the metaproteomic approach has been successfully applied to a wide gamut of samples, including acid mine drainage biofilms, waste and ocean waters, and soil, as well as the most diverse plant- and animal/human-associated environments, from leaf to gut [2-5].

Although computational analysis is widely recognized as the most impacting challenge in shotgun metaproteomics, the optimization of sample preparation procedures preceding mass spectrometry (MS) identification deserves special attention. The effectiveness of a method suitable for the shotgun proteomic analysis of a complex metaproteome relies essentially on three main steps: i) protein extraction: a comprehensive protein complement of the entire microbial community needs to be extracted in an efficient, unbiased, and reproducible way; ii) cleanup: most detergents used for extraction and/or interfering environmental compounds need to be removed before proteins are digested into peptides; and iii) pre-fractionation: peptides (and/or proteins) need to be adequately separated prior to MS detection, in order to reduce sample complexity and increase analysis depth. Furthermore, very complex and heterogeneous samples (such as intestinal contents) may also require a preliminary separation step to selectively enrich in microbial cells and reduce host material. Given the relative youth of metaproteomics, as well as the huge variability among microbial community samples and within community members in terms of biochemical features and structural complexity, significant research efforts are still required to improve, optimize, and standardize sample preparation workflows for metaproteome analysis.

Concerning protein extraction, a wide assortment of chemical and physical methods (alone or variously combined) have been described to date as suitable to lyse microbial cells from environmental samples, including the use of buffers containing one or more components among detergents (sodium dodecyl sulfate (SDS), CHAPS, Triton X-100) [1,6,7], chaotropic agents (urea, guanidine hydrochloride) [1,8], reducing agents (dithiothreitol (DTT), tributylphosphine) [9], and other organic/inorganic compounds (phenol, NaOH) [10-12], as well as thermal treatments (boiling, freeze-thawing, snap-freezing) [13-15], mechanical disruption (French press, bead-beating, grinding) [1,10,16,17], and sonication [18,19]. Since Gram-positive bacteria, Gram-negative bacteria, and fungi show tremendous structural differences and hence diverse extents of susceptibility to each protein extraction method, an optimized protocol should maximize extraction yield, avoiding a selective depletion of species with higher resistance to lysis.

After proteins have been extracted from the microbial community, compounds which may hamper enzymatic digestion, liquid chromatography (LC) separation, or MS analysis have to be removed. This goal is classically achieved by protein precipitation, which can be accomplished by adding, for instance, trichloroacetic acid, acetone, or ammonium acetate/methanol to the protein extract [7,11,12,20]; the protein pellet is then resuspended in a buffer compatible with the subsequent steps. However, significant (and possibly selective) losses due to protein aggregation can likely occur [21,22]. Another effective option is to perform one-dimensional electrophoresis (1-DE) protein separation followed by in-gel digestion of the extracted proteins, which allows both the entrapment of interfering compounds within the gel matrix and the sample fractionation into gel slices [17,23-25]. Unfortunately, although efficient, this method is labor-intensive and time-consuming, and reproducibility may not be optimal [26]. A recent alternative is represented by the filter-aided sample preparation (FASP), in which sample cleanup and enzymatic cleavage take place in a molecular weight cutoff centrifuge filter [27]. This procedure has been recently applied with success to microbiome samples and was demonstrated to outperform several competing methods especially for low protein amounts [19,20].

Furthermore, sample complexity needs to be reduced in order to improve the extent of information achievable by shotgun MS analysis. This has been attained in previous metaproteomic studies by carrying out a fractionation at the protein (mainly by 1-DE and GELFrEE approaches) [17,20,28] and/or peptide level (most commonly by means of two-dimensional liquid chromatography (2D-LC)) [8,15,29]. However, each additional fractionation step implies a corresponding increase in the amount of starting material, laboratory effort, and/or MS measuring time required, as well as increasing challenges in analytical reproducibility. In particular, 2D-LC tandem mass spectrometry (MS/MS), notwithstanding its very remarkable analysis depth, is technically demanding and, above all, requires extremely long times for a single sample to be analyzed (22 h in a typical experimental setting for metaproteome samples) [8,30]. Recently, a straightforward approach based on single-run nanoLC-MS/MS has been described, enabling the identification of several thousands of proteins per run from different kinds of samples [30-34].

In this work, we present an optimized analytical pipeline for analysis and characterization of metaproteomes, which comprises bead-beating/freeze-thawing for protein extraction, FASP for cleanup and digestion, and single-run nanoLC-MS/MS for peptide separation and identification. First, the pipeline performance was evaluated using mock microbial mixtures, in order to test its efficiency, sensitivity, and dynamic range. Then, the pipeline was applied to mine the mouse stool metaproteome, with the aim of validating its ability to provide reliable, reproducible, and deep taxonomic and functional information from a complex microbiome.

## Results and discussion

### Preliminary protein extraction optimization

**Figure 1:**
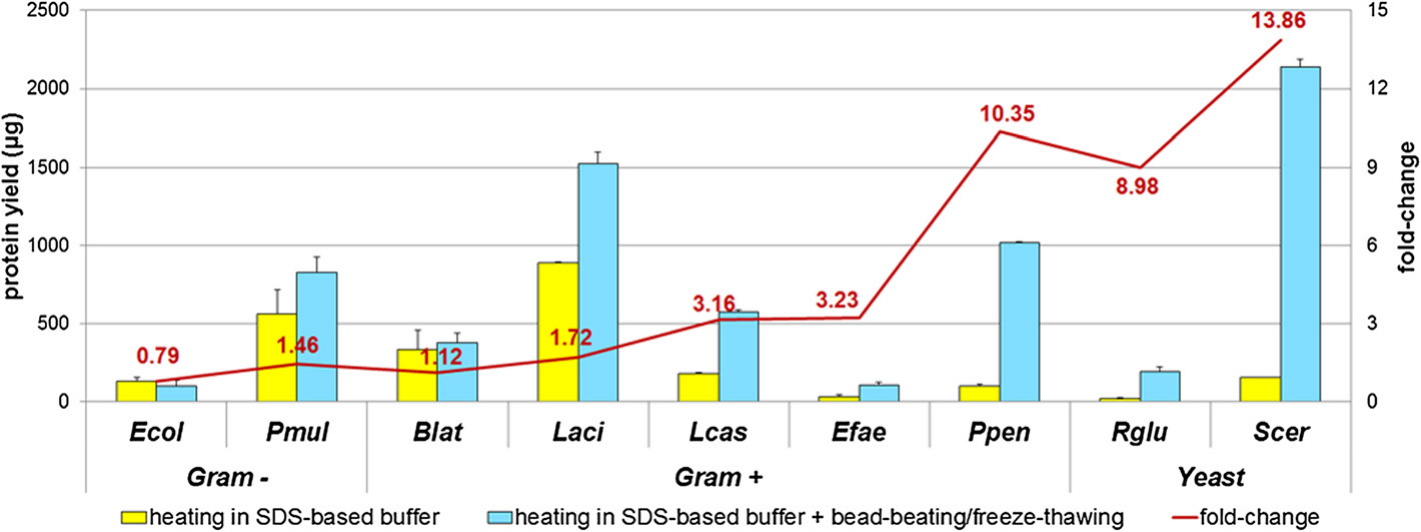
**Protein yields with two different extraction methods.** Histogram showing protein quantification results (mean of three replicates; *error bars* indicate standard deviation). Fold-change values were calculated by dividing the yield obtained with the second method (heating in SDS-based buffer + bead-beating/freeze-thawing) by the yield obtained with the first method (heating in SDS-based buffer). Abbreviations: Ecol: *E. coli*; Pmul: *P. multocida*; Blat: *B. laterosporus*; Laci: *L. acidophilus*; Lcas: *L. casei*; Efae: *E. faecalis*; Ppen: *P. pentosaceus*; Rglu: *R. glutinis*; Scer: *S. cerevisiae*.

First, we selected nine microorganisms (seven bacterial and two eukaryotic strains; see Additional file 1: Table S1 for details) exhibiting very different structural features, with the aim of mimicking the various degrees of resistance to lysis which can be found in a microbial community. In particular, our initial purpose was to assess if, and to what extent, the addition of bead-beating and freeze-thawing treatments would impact on protein extraction yield, compared to simple extraction by heating in a SDS-based buffer. Each microorganism was therefore subjected to protein extraction according to both methods (*N* = 3 replicates per method), and the extraction yield was determined by protein quantification using the 2-D Quant Kit. Results shown in Figure 1 demonstrate that the combination of bead-beating and freeze-thawing dramatically increases protein extraction yields from yeasts (up to 14-fold) and Gram-positive bacteria (up to 10-fold), without detrimental effects on Gram-negative bacteria and with low variability among replicates. This extraction procedure is therefore useful to maximize protein extraction from microbial species which are resistant to cell wall lysis, in line with previous data regarding DNA and protein extraction from microbial pure cultures and complex microbial communities [17,35-38].

### Overview of pipeline steps and study design

**Figure 2:**
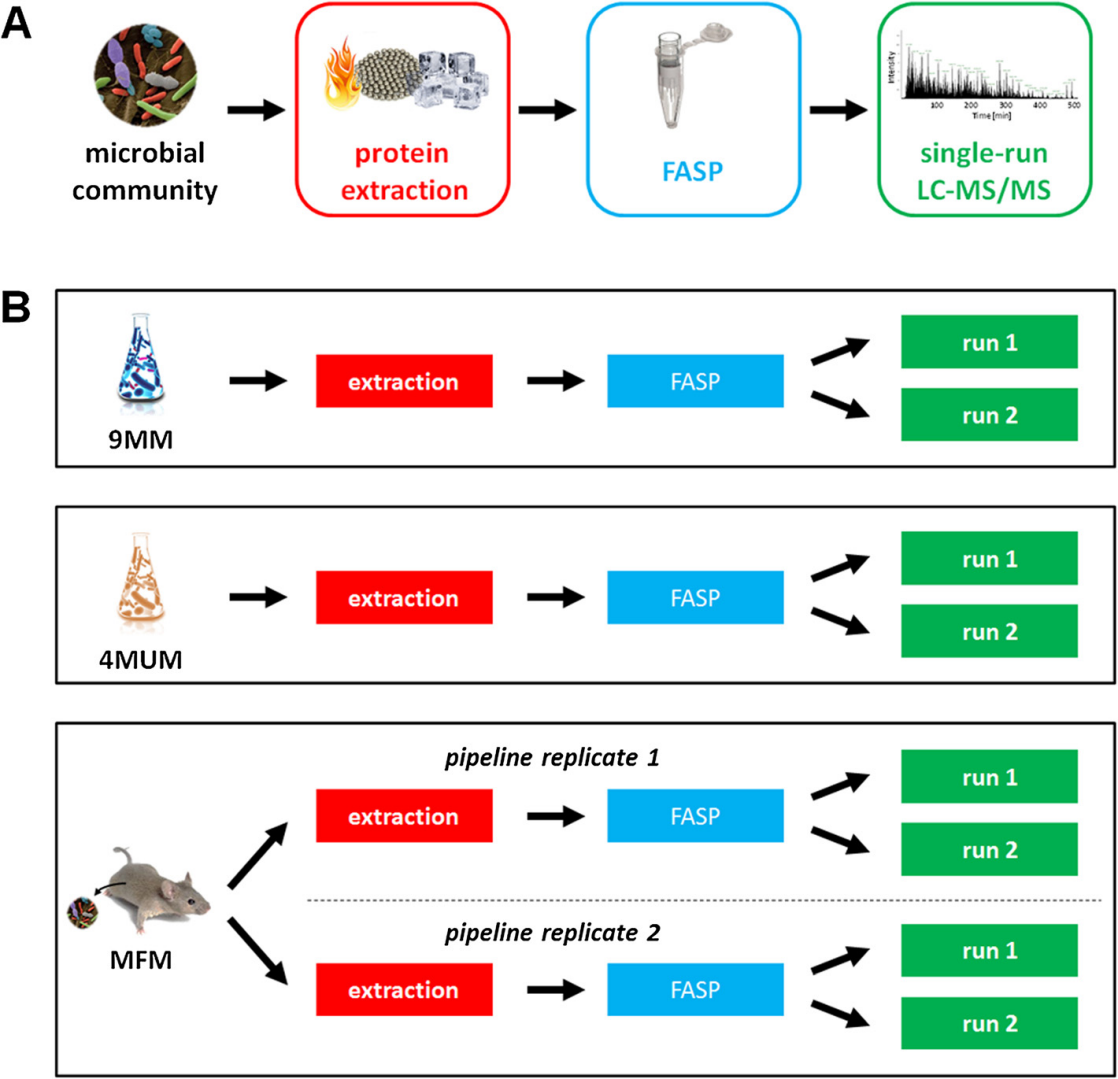
**Overview of pipeline steps and study design. (A)** Schematic representation of the experimental steps comprised in the pipeline presented in this study. **(B)** Schematic representation of the experimental design used in this study.

The pipeline for metaproteome analysis presented in this work, as detailed below in the “Methods” section and illustrated in Figure 2A, consists of three main steps: i) proteins are extracted from microbial community samples by heating in SDS-based buffer followed by bead-beating/freeze-thawing steps (approximately 1.5 h); ii) protein extracts are cleaned up and digested on-filter according to the FASP procedure (8 h to overnight); and iii) peptide mixtures are subjected to single-run LC-MS/MS analysis using an 8-h gradient.

The experimental design used in this study is schematized in Figure 2B. Initially, the pipeline performance was evaluated using two lab-assembled microbial mixtures of known composition (nine-microorganism mixture (9MM) and four-microorganism unbalanced mixture (4MUM), see below for details), in order to test its efficiency, sensitivity, and dynamic range. Then, the pipeline applicability to complex microbiome samples was validated by analyzing a “real-world” sample, namely the murine fecal microbiome (MFM). As depicted, the reproducibility of the LC separation step (referred to as “run reproducibility”) was evaluated for all the samples analyzed by comparing two replicate LC-MS/MS runs, whereas the reproducibility of the entire pipeline (referred to as “pipeline reproducibility”) was evaluated only for the MFM by dividing the same stool sample into two portions and performing two independent “whole pipeline” replicates.

### Pipeline evaluation: mock microbial mixtures

#### Nine-microorganism mixture

A first microbial mixture, named 9MM, was assembled by mixing the nine microbes previously used for the protein extraction optimization. The proteins extracted from the 9MM were digested according to the FASP procedure, and the peptide mixture obtained was analyzed in duplicate by single-run LC-MS/MS (Figure 2B). MS spectra were searched against a matched genomic database, containing all open reading frames (ORFs) achieved upon experimental genome sequencing of the nine individual strains (see “Methods” section for details). Throughout the manuscript, the term “peptides” is referred to non-redundant peptide sequences, whereas “peptide-spectrum matches” (PSMs) is referred to all peptide sequences identified, including those redundantly detected.

**Figure 3:**
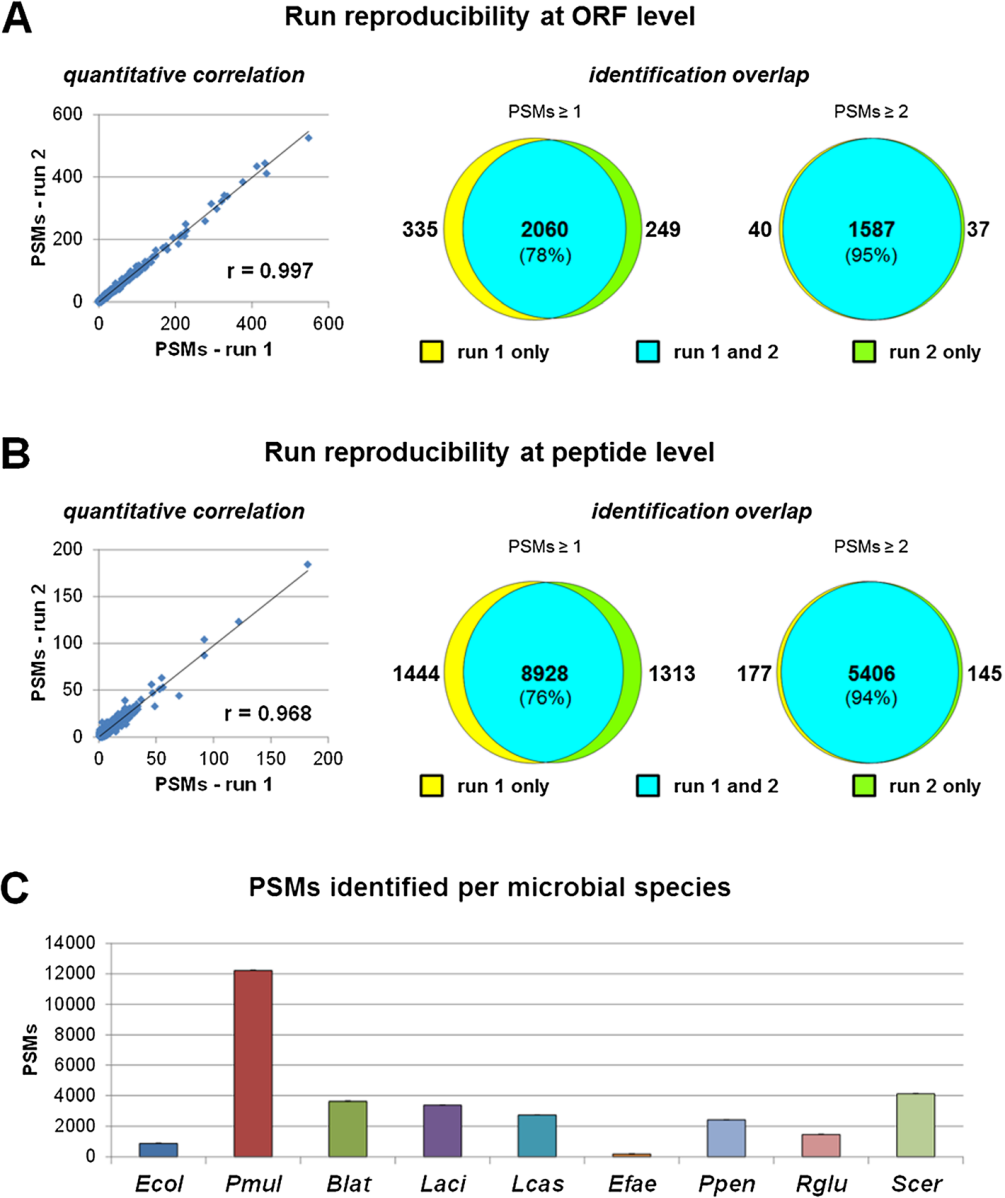
**Pipeline evaluation using 9MM. (A)** Analysis of run reproducibility at the ORF level. Left: scatter plot illustrating the correlation between the number of PSMs detected in two runs (*r* = Pearson correlation coefficient); each point in the plot represents an identified ORF. Right: Venn diagrams depicting the overlap of ORF identifications between the two runs (left diagram, all identified ORFs; right diagram, ORF identified with at least two PSMs; percentage of overlap in brackets). **(B)** Analysis of run reproducibility at peptide level. Left: scatter plot illustrating the correlation between the number of PSMs detected in the two runs (*r* = Pearson correlation coefficient); each point in the plot represents an identified peptide. Right: Venn diagrams depicting the overlap of peptide identifications between the two runs (left diagram, all identified peptides; right diagram, peptide identified with at least two PSMs; percentage of overlap in brackets). **(C)** Quantitative estimation of microbial species abundance according to the number of PSMs identified. For abbreviations of microbial strains, see caption of Figure 1.

The application of the analytical pipeline to the 9MM led to the MS identification of almost 29,000 PSMs on average per run, belonging to over 2,000 different ORFs and corresponding to over 10,000 non-redundant tryptic peptide sequences (further details are given in Additional file 2: Table S2). Furthermore, the single-run LC separation exhibited a high run reproducibility, both in quantitative (Pearson correlation coefficient *r* = 0.997 and 0.968 at the ORF and peptide level, respectively; Figure 3A,B, left) and qualitative terms (78% and 76% identification overlap between runs at the ORF and peptide level, respectively, raising to 95% and 94%, respectively, when considering identifications with at least two PSMs; Figure 3A,B, right). Peptide sequences from ORFs belonging to all nine species, including Gram-positive bacteria and yeasts, were consistently detected (numbers of PSMs per species are reported in Figure 3C).

In order to evaluate the ability of the pipeline to extract and identify membrane proteins, the percentage of identified ORFs containing transmembrane domains (TMDs) was also estimated, corresponding to 7.2% on average per run, with 4.8% with a single TMD and 2.4% with two or more TMDs (i.e., about 60 multipass membrane proteins detected per run).

#### Four-microorganism unbalanced mixture

Four bacterial strains were then selected to assemble a simpler mock mixture, named 4MUM. In this case, the amount of bacterial cells was accurately measured, and bacteria were mixed in unbalanced proportions (specifically, 10^10^ colony-forming units (CFUs) of *Enterococcus faecalis*, 10^8^ CFUs of *Escherichia coli* and *Pasteurella multocida*, respectively, and 10^6^ CFUs of *Lactobacillus acidophilus*), with the purpose of testing sensitivity and linearity of the method in relation to the bacterial cell amount. The 4MUM underwent the same analytical pipeline used for the 9MM (Figure 2B).

**Figure 4:**
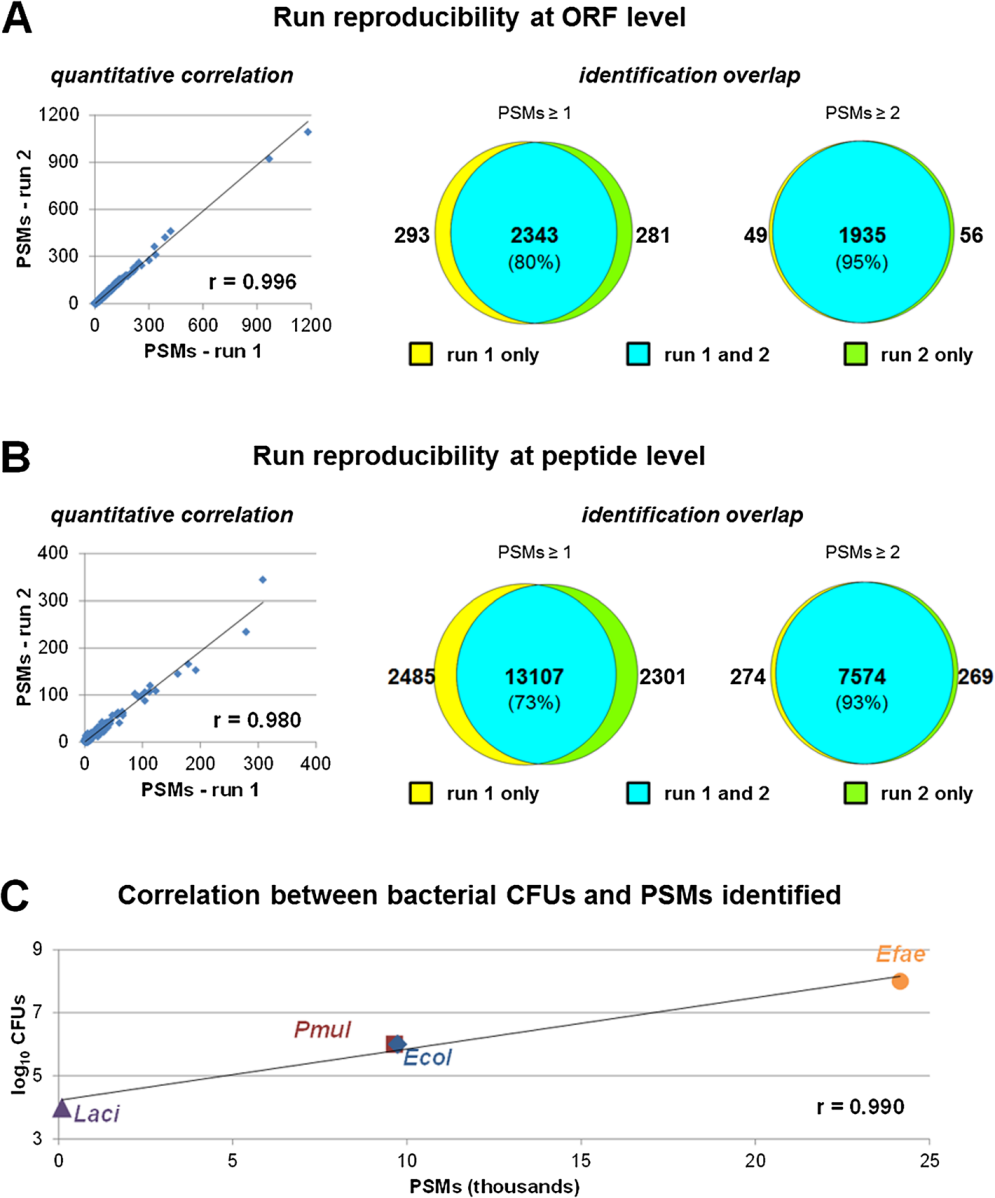
**Pipeline evaluation using 4MUM. (A)** Analysis of run reproducibility at the ORF level. Left: scatter plot illustrating the correlation between the number of PSMs detected in the two runs (*r* = Pearson correlation coefficient); each point in the plot represents an identified ORF. Right: Venn diagrams depicting the overlap of ORF identifications between the two runs (left diagram, all identified ORFs; right diagram, ORF identified with at least two PSMs; percentage of overlap in brackets). **(B)** Analysis of run reproducibility at the peptide level. Left: scatter plot illustrating the correlation between the number of PSMs detected in two runs (*r* = Pearson correlation coefficient); each point in the plot represents an identified peptide. Right: Venn diagrams depicting the overlap of peptide identifications between the two runs (left diagram, all identified peptides; right diagram, peptide identified with at least two PSMs; percentage of overlap in brackets). **(C)** Scatter plot illustrating the correlation between the number of bacterial CFUs (namely, 10^10^ CFUs of *E. faecalis*, 10^8^ CFUs of *E. coli* and *P. multocida*, respectively, and 10^6^ CFUs of *L. acidophilus*) and the corresponding number of identified PSMs. For abbreviations of microbial strains, see caption of Figure 1.

Concerning standard identification statistics, almost 41,000 PSMs on average per run could be detected, belonging to over 2,600 different ORFs and corresponding to over 15,000 non-redundant tryptic peptide sequences (Additional file 2: Table S2). In addition, a high level of reproducibility between LC-MS/MS runs was confirmed, with *r* = 0.996 and 0.980 at the ORF and peptide level, respectively (Figure 4A,B, left), and 80% and 73% identification overlap at the ORF and peptide level, respectively (raising to 95% and 93%, respectively, when considering identifications with at least two PSMs; Figure 4A,B, right).

More interestingly, a mean of 95 PSMs per run (corresponding to 56 non-redundant tryptic peptides sequences, belonging to 37 different ORFs) were assigned to the less abundant species (*L. acidophilus*). Considering that the total amount of protein extracted from the 4MUM was nearly 350 μg, and the amount of peptide mixture actually loaded per run was 4 μg (therefore, about the 90th part of the initial 10^6^ *L. acidophilus* CFUs), the sensitivity of the pipeline can be estimated as equal to (or lower than) 10^4^ CFUs. A linear dynamic range was therefore observed from 10^4^ to 10^8^ CFUs, with detectable CFU values spreading along 5 orders of magnitude, wider than previously observed [2]. In fact, the number of PSMs assigned to each bacterial species was highly correlated (*r* = 0.990) to the bacterial cell amount (expressed as log_10_ CFUs; Figure 4C).

Finally, 14.3% of the identified ORFs contained at least one TMD (8.6% with two or more TMDs), corresponding to over 220 multipass membrane proteins found per run.

#### Considerations on results achieved using mock microbial mixtures

On the whole, the data achieved with 9MM and 4MUM samples are largely comparable, in qualitative and quantitative terms, to the results obtained using LC gradients of similar length in previous works in which simpler samples were analyzed [30-33]. It has to be noted, however, that the 4MUM provided a higher number of identifications, particularly membrane protein sequences, although starting from the same peptide load (4 μg) and number of MS spectra (around 70,000) of the 9MM. This might be due to the lower genome coverage of the eukaryotic microorganisms, comprised in the 9MM and not in the 4MUM, as well as to the possible presence in the 9MM of a higher amount of non-peptidic ionizing molecules (e.g., lipids and secondary metabolites) which could not be identified upon standard MS analysis.

The duration of the LC gradient (8 h) was determined based on the literature [30,31] as well as on the results of preliminary optimization experiments performed in our lab (data not shown). Among the latter, a comparative experiment was carried out by analyzing the same peptide mixture obtained from the 9MM sample using both 4- and 8-h LC gradients. In this experiment, the 8-h gradient provided 35% and 45% more non-redundant ORF and peptide identifications, respectively, compared to the 4-h gradient. It is worth noting that comparable results may be also obtained by using gradients shorter that 8 h with longer columns or particles of smaller size [32,33], and that in every lab, the whole LC configuration should be conveniently optimized depending on the particular system used and the desired/required analysis throughput.

### Pipeline validation: murine fecal microbiome

The murine fecal microbiome was chosen to validate the reliability and suitability of the pipeline when applied to complex metaproteome samples. Stool was preliminary subjected to differential centrifugation (as performed in earlier gut metaproteomics studies [8,39]) in order to produce a microbial pellet, which was then subjected to the pipeline previously evaluated on the mock microbial mixtures. As illustrated in Figure 2B, two portions from the same stool microbial pellet were processed in parallel as technical replicates (from protein extraction to FASP) and, for each replicate, two separate LC-MS/MS analyses were run, with the aim of evaluating both the pipeline and run reproducibility. The MS spectra were finally searched against a matched metagenomic database, containing all ORFs achieved upon experimental sequencing of the whole fecal metagenome plus the mouse and soybean proteomes, as well as selected fungal and archaeal sequences (see “Methods” section for details).

#### Identification statistics and evaluation of pipeline and run reproducibility

The application of the analytical pipeline to the MFM led to the identification of up to over 26,000 PSMs per run, belonging to 9,000 different ORFs and corresponding to 13,000 non-redundant tryptic peptide sequences (Additional file 2: Table S2). On the whole, over 18,000 non-redundant tryptic peptide sequences could be detected, of which 93% of microbial origin. Concerning protein topology/localization, 11% of the identified ORFs contained at least one TMD (5% with two or more TMDs), corresponding to over 200 multipass membrane proteins found per run.

**Figure 5:**
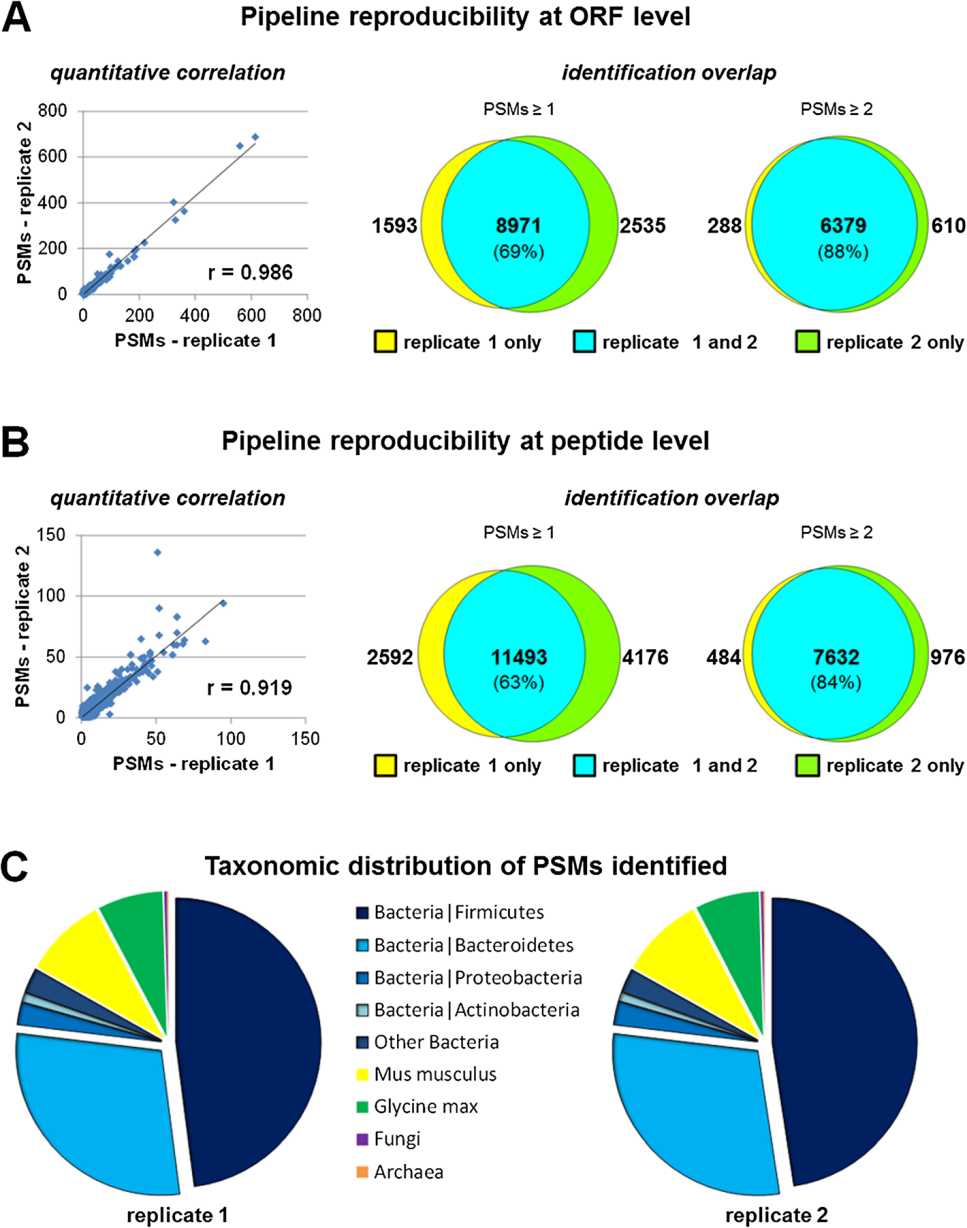
**Validation of the pipeline performance and reproducibility by murine fecal metaproteome analysis. (A)** Analysis of pipeline reproducibility at the ORF level. Left: scatter plot illustrating the correlation between the number of PSMs detected in the two replicates (*r* = Pearson correlation coefficient); each point in the plot represents an identified ORF. Right: Venn diagrams depicting the overlap of ORF identifications between the two replicates (left diagram, all identified ORFs; right diagram, ORF identified with at least two PSMs; percentage of overlap in brackets). **(B)** Analysis of pipeline reproducibility at the peptide level. Left: scatter plot illustrating the correlation between the number of PSMs detected in the two pipeline replicates (*r* = Pearson correlation coefficient); each point in the plot represents an identified peptide. Right: Venn diagrams depicting the overlap of peptide identifications between the two pipeline replicates (left diagram, all identified peptides; right diagram, peptide identified with at least two PSMs; percentage of overlap in brackets). **(C)** Pie charts illustrating the percentage distribution of the identified PSMs according to LCA taxonomy (left: replicate 1; right: replicate 2).

As far as pipeline reproducibility is concerned, PSM values between replicates showed a good correlation (*r* = 0.986 and 0.919 at the ORF and peptide level, respectively, as illustrated in Figure 5A,B, left), while identification overlap at the ORF and peptide level was 69% and 63%, respectively, raising to 88% and 84%, respectively, when considering identifications with at least two PSMs (Figure 5A,B, right). The PSM values of a pipeline replicate were calculated as the sum of the PSM values obtained in the two runs carried out for that pipeline replicate. On the other hand, the following average values were measured in respect to run reproducibility: *r* = 0.971 and 0.866 at the ORF and peptide level, respectively; 63% overlap for ORFs and 56% for peptides, increasing up to 85% and 80% for ORFs and peptides, respectively, when considering identifications with at least two PSMs (Additional file 3: Figure S1).

#### Taxonomic distribution of the murine fecal metaproteome

Stool metagenomic ORFs (used as database sequences) were classified according to the lowest common ancestor (LCA) approach using the metagenomic analysis server MG-RAST [40]. In order to take a quantitative picture of the metaproteomic taxonomic distribution, the abundance of a given taxon was measured as proportional to the number of identified PSMs mapping on all ORFs classified as belonging to that taxon. As apparent from the pie charts of Figure 5C, the distribution obtained by analyzing the two pipeline replicates was almost identical. According to LCA results, a remarkable microbial diversity could be observed in the MFM sample. In fact, peptide sequences were classified as belonging to the following numbers of different microbial taxa (as detailed in Additional file 4: Table S3): 29 phyla, 49 classes, 84 orders, 148 families, 317 genera, and 683 species. When considering only the peptide sequences assigned to bacteria, the most represented phyla were *Firmicutes* (58%) and *Bacteroidetes* (36%), with 24 different phyla detected in total (compared to 17, as recently reported by Del Chierico et al. [41]), nine of which exceeding a 0.1% abundance threshold (as recently described in a meta-analysis of the mouse core gut microbiome by 16S rRNA gene sequencing with a comparable abundance threshold [42]). Going to lower taxonomic levels, two single classes accounted together for about 90% of the MFM (*Clostridia* and *Bacteroidia*), while a higher diversity could be observed at a family level, with *Lachnospiraceae* (25%), *Porphyromonadaceae* (15%), *Clostridiaceae* (15%), *Bacteroidaceae* (10%), *Prevotellaceae* (7%), and *Ruminococcaceae* (7%) exceeding a 5% threshold. The dominating genera were *Clostridium* (17%), *Bacteroides* (12%), *Tannerella* (9%), and *Prevotella* (7%), with over 50 genera and 168 species being above the 0.1% abundance threshold. Moreover, the MFM analysis led to the identification of peptide sequences belonging to the host (*Mus musculus*, 9%), to the soybean-based feed (*Glycine max*, 7%), to the *Fungi* kingdom (0.3%), and to the *Archaea* superkingdom (0.15%).

#### Functional characterization of the murine fecal metaproteome

Mouse fecal metaproteome results were further analyzed to carry out a functional characterization of the microbiome. Specifically, according to the Universal Protein Resource Knowledgebase (UniProtKB) protein family classification, 273 protein families of microbial origin were identified in the MFM sample. The top 25 microbial protein families are displayed in Table 1, whereas Additional file 5: Table S4 presents the complete list of protein families detected (along with the related LCA taxonomic information). The identified families covered a wide range of enzymatic, transport, and signaling functions; among them, the TonB-dependent receptor family (TBDRF, accounting for 255 different ORFs identified by metaproteomics in this study) was chosen as a representative example. The TBDRF proteins belong to a transport system enabling the active uptake of nutrients (mainly iron complexes and vitamin B12, but also nickel and carbohydrates) across the outer membrane of Gram-negative bacteria [43,44]. An increasing clinical significance has been recognized to this transport system, since survival of commensal and pathogenic bacteria depends on the ability to compete for micronutrients, such as iron [45,46]. In order to shed light on the particular members of the mouse gut microbiota actively expressing this particular type of outer membrane receptors, taxonomic information was assigned to each TBDRF member according to an LCA approach (Additional file 6: Table S5). As a result, over 99% of PSMs assigned to the TBDRF were related to the phylum Bacteroidetes, which appears to be the most clearly involved in this function within the mouse gut microbiota. Moreover, 32 and 12 non-redundant peptide sequences were assigned to the ExbB/tolQ and ExbD/tolR families, which form the membrane complex responsible for energy production in the TonB-dependent system. Noteworthy, the expression of TBDRF genes by *Bacteroides* species has been found actively upregulated in an experimental rat model of chronic colitis [47]. Hence, the ability to extensively identify TBDRF proteins might be of key interest to functionally characterize the gut microbiome and its cross talk with the host immune system.

**Table 1:** **Top 25 microbial protein families detected in the mouse stool sample**

**UniProt protein family**	**ORFs** ^**a**^	**Peptides** ^**a**^	**PSMs**
TonB-dependent receptor family	255	345	1,433
GTP-binding elongation factor family	235	379	4,676
NifJ family	209	278	1,699
ATPase alpha/beta chains family	171	213	1,168
Chaperonin (HSP60) family	138	200	1,294
Glyceraldehyde-3-phosphate dehydrogenase family	110	153	1,186
ABC transporter superfamily	108	119	420
Glu/Leu/Phe/Val dehydrogenases family	89	127	637
Class-II aminoacyl-tRNA synthetase family	84	95	356
RNA polymerase beta chain family	74	93	416
Phosphoglycerate kinase family	72	92	489
Enolase family	69	105	765
Glycogen phosphorylase family	67	95	482
Heat shock protein 70 family	63	100	646
RNA polymerase beta′ chain family	63	86	440
Binding-protein-dependent transport system permease family	54	59	241
Class-I aminoacyl-tRNA synthetase family	54	61	217
Phosphoenolpyruvate carboxykinase family	54	78	402
Polyribonucleotide nucleotidyltransferase family	50	64	338
LDH/MDH superfamily	49	69	325
Triosephosphate isomerase family	49	68	373
Phosphohexose mutase family	46	56	181
Acyl-CoA dehydrogenase family	44	59	316
RNA polymerase alpha chain family	43	67	283
GPI family	41	49	211

^a^non-redundant.

**Figure 6:**
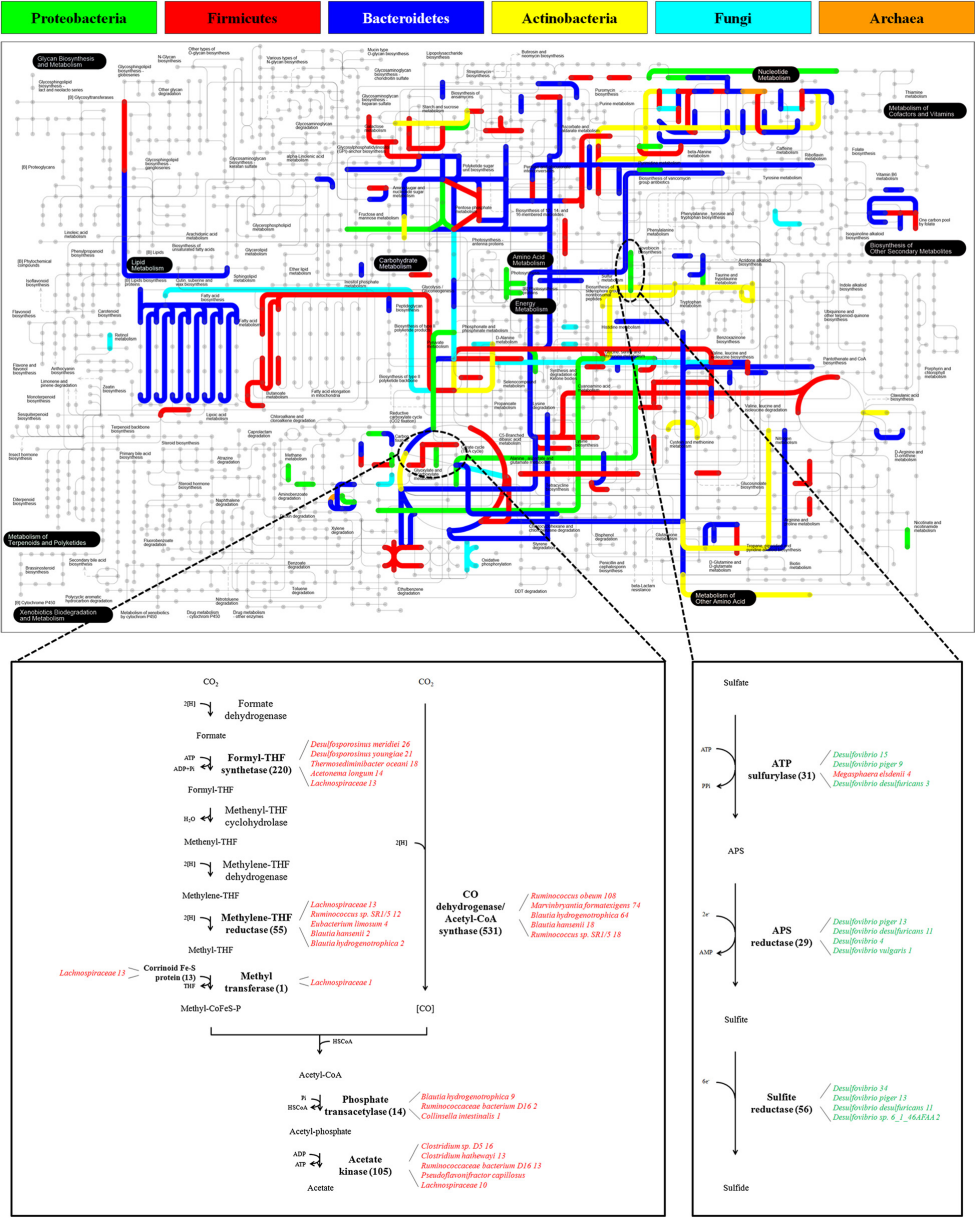
**Metabolic pathway analysis of the murine fecal metaproteome.** Top: distribution of the identified proteins belonging to the six main microbial kingdoms/phyla into metabolic pathways using iPATH. The upper legend explains the correspondence between colors and taxa. Bottom: detail of the Wood-Ljungdahl pathway (left) and of the dissimilatory sulfate reduction pathway (right). The proteins identified in this study are in bold type and their corresponding overall PSMs in brackets, while the adjacent text in italic illustrates the corresponding taxonomic assignments based on the LCA approach (phylum/kingdom-related color according to the upper legend) and listed according to the number of PSMs. Complete details are given in Additional file 7: Table S6. Abbreviations: *ADP* adenosine-diphosphate, *APS* adenosine-5’-phosphosulfate, *THF* tetrahydrofolate.

Furthermore, microbial protein identities were uploaded into iPATH [48] with the aim of mapping proteins into metabolic pathways. As shown in the metabolic map in Figure 6 (top), enzymes included in numerous microbial metabolic pathways were successfully identified within the MFM metaproteomic dataset, with a well-balanced contribution of all the main bacterial phyla, as well as of the fungal part (different colors in the image indicate a different taxonomy). Two representative pathways were selected for further analyses, in view of their importance in gut metabolism and specifically in the oxidation of the hydrogen generated during the microbial fermentation of dietary macromolecules. The first is the Wood-Ljungdahl pathway, in which hydrogen and carbon dioxide are converted into acetic acid; this pathway is used by acetogenic prokaryotes as their main mechanism for energy conservation, as well as for acetyl-coenzyme A (CoA) synthesis from carbon dioxide (Figure 6, bottom left) [49-51]. The second is the dissimilatory sulfate reduction, in which sulfate is the terminal electron acceptor for anaerobic respiration, with the concomitant production of hydrogen sulfide by sulfate-reducing bacteria (SRB; Figure 6, bottom-right) [52-54]. In the human gut microbiota, where this pathway is used mostly by SRB belonging to Deltaproteobacteria, the impact of sulfide in health and disorders of the gut colonic mucosa still requires in-depth investigations [55]. The analysis of the MFM metaproteome enabled to map both pathways with a remarkable coverage, with the consistent assignment of each enzymatic function to a specific taxa, in most cases down to the species level. Concerning the Wood-Ljungdahl pathway, several members of the family *Lachnospiraceae* (including species from the genera *Ruminococcus*, *Blautia*, and *Marvinbryantia*) were found to be primarily involved in the main reactions of the pathway. As regards the dissimilatory sulfate reduction pathway, almost all peptide identifications were attributed to the genus *Desulfovibrio* (*Desulfovibrio piger* being the main species), well-known to be the most abundant among gut SRB [56]. Complete details about taxonomic assignments concerning these two pathways are given in Additional file 7: Table S6. Therefore, such examples demonstrate that the application of the proposed pipeline may provide deep and precise information concerning the particular role exerted by the different members of a microbiota within complex metabolic pathways.

#### Considerations on results achieved analyzing the mouse fecal metaproteome

To the best of our knowledge, the MFM data presented in this work represent one of the largest fecal metaproteome dataset published to date. Earlier mouse gut metaproteome studies reported 1,760 microbial proteins identified from eight mouse cecal samples (0.6% false discovery rate (FDR)) [57] and 997 microbial proteins identified from 18 newborn mouse cecal samples (1% FDR) [41] (no information about the number of non-redundant peptide identification was given in both papers); further, the analysis of rat feces and intestinal contents has led to no more than 2,802 bacterial proteins from 20 samples (5% FDR) [23]. Taking into account also gut metaproteome studies on human samples (or humanized animal models), the best results in terms of number of proteins/peptides detected were achieved by combining in-solution digestion and 2D-LC separation, with up to 9,000 microbial peptides and 4,000 proteins (with at least two unique peptides) identified per sample [8,39,58], even if applying FDR thresholds less stringent than in this work and using much longer LC gradients. On the other hand, the application of 1-DE protein fractionation followed by in-gel digestion and LC-MS/MS [17,28] has reached less valuable results and, above all, is considerably more time-consuming and labor-intensive compared to the pipeline described here.

It is worth noting that the differential centrifugation pretreatment, although often used in gut metaproteomics, should not be considered as a constitutive step of the pipeline presented here. In fact, this pipeline was not thought to be exclusively suited for fecal samples, and most of all, stool can also be subjected to protein extraction without a preliminary treatment aimed at enriching in microbial cells (e.g., by direct homogenization in extraction buffer). We have preliminary evidences that our pipeline can be successfully used with non-pretreated fecal samples, with yields and data quality comparable to those achieved upon differential centrifugation, except for an obvious higher percentage of host proteins (unpublished data). The impact of different stool pretreatment methods in gut metaproteomics is actually a very interesting topic; for instance, it remains to be verified whether differential centrifugation, especially when dealing with frozen fecal material, might lead or not to selective losses of certain microbial or protein categories. However, this should deserve specific research efforts lying outside the specific aim of this work.

In addition, it has to be considered that the approach used for protein identification and data analysis might have a dramatic impact in metaproteomics, as demonstrated and discussed elsewhere [59,60]. In this work, we chose to use a matched (meta)genomic database (i.e., obtained upon DNA sequencing of the same microbial strains or microbial communities which are analyzed by metaproteomics), in order to maximize “compatibility” between theoretical and experimental spectra for MS identification and to minimize issues related to the large size of public databases, as well documented and successfully applied in previous metaproteomic studies concerning the gut microbiome [28,39]. In particular, raw reads (i.e., not assembled into contigs) were employed for generating the metagenomic database, according to the results shown by previous works [61]. Furthermore, in view of the apparently low amount of fungal and archaeal sequences within the metagenome (which may be due to biases in DNA extraction, or a consequence of an actual under-representation of such microbial members within the MFM), we decided to append additional UniProtKB entries belonging to fungal genera previously reported as being present in the (mouse) gut microbiome and to methanogenic Archaea [62,63]. As a matter of fact, we can expect that progresses and refinements in metaproteome bioinformatics in the near future might lead to obtain improved and significantly different results with the analytical pipeline described in this work.

## Conclusions

This work presents a straightforward and efficient pipeline for metaproteome analysis. The overall procedure can be accomplished in a minimum of approximately 18 h, while the best performing method developed to date (in-solution digestion coupled to 2D-LC-MS/MS) requires at least 22 h solely for the 2D-LC separation. This optimized pipeline enables the identification of proteins from complex microbial assortments with different cell types and structural features (including fungi), with high reproducibility and reaching a sensitivity down to 10^4^ bacterial CFUs. When applied to fecal samples, the approach proposed in this work has led to the identification of proteins belonging to over 600 different microbial species and mapping to over 250 functionally relevant protein families, with a significant level of detail on the metabolic pathways actively functioning in the gut microbiota. In keeping with this, the pipeline described here may be successfully used for the in-depth and time-effective characterization of complex microbiomes.

## Methods

### Samples

#### Microbial strains and mock mixtures

The characteristics of the microbial strains used in this study are presented in Additional file 1: Table S1 and further detailed elsewhere [60]. *Escherichia coli* and *Enterococcus faecalis* were available in the bacterial collection of the Department of Biomedical Sciences, University of Sassari, whereas *Lactobacillus casei*, *Lactobacillus acidophilus*, *Pediococcus pentosaceus*, and *Saccharomyces cerevisiae* were available in the laboratories of Porto Conte Ricerche. *Pasteurella multocida* was kindly provided by Dr. Gavino Marogna (Istituto Zooprofilattico Sperimentale della Sardegna), *Rhodotorula glutinis* by Prof. Ilaria Mannazzu (Department of Agricultural Sciences, University of Sassari), and *Brevibacillus laterosporus* by Dr. Luca Ruiu (Bioecopest Srl). Microbial cultures were grown at 37°C to a stationary phase using the appropriate standard medium for each microorganism. The microbial cultures were divided into 1-ml aliquots, washed three times in phosphate-buffered saline (PBS), pelleted, and stored at −80°C until use.

A 9MM was then assembled by suspending sequentially each microbial pellet in a single aliquot of the extraction buffer described below, immediately before protein extraction.

Four bacteria (namely, *E. coli*, *E. faecalis*, *L. acidophilus*, and *P. multocida*) were selected for further analyses. After overnight culture, each bacterial strain was subjected to accurate CFU counting, divided into three aliquots (corresponding to 10^10^, 10^8^, and 10^6^ CFUs, respectively), pelleted, washed three times in PBS, dried, and stored at −80°C until use. A 4MUM was then assembled by merging a pellet corresponding to 10^10^ CFUs of *E. faecalis*, two pellets corresponding to 10^8^ CFUs of *E. coli* and *P. multocida*, respectively, and a pellet corresponding to 10^6^ CFUs of *L. acidophilus*.

#### Murine fecal sample

A fecal sample (weight approximately 100 mg), collected from a 37-weeks old NOD mouse and immediately stored at −80°C until use, was kindly provided by Dr. Michael Silverman (Mathis-Benoist Laboratory, Department of Microbiology and Immunobiology, Harvard Medical School). After thawing at 4°C, the fecal sample was subjected to differential centrifugation to enrich in microbial cells, according to Apajalahti et al. [64] with minor modifications. Briefly, the sample was resuspended in 10 ml of PBS, vortexed, shaken in a tube rotator for 45 min, and subjected to low-speed centrifugation at 500 × *g* for 5 min to eliminate gross particulate material; the supernatant was carefully transferred to a clean polycarbonate centrifuge bottle (Beckman Coulter, Brea, CA, USA) and kept at 4°C, whereas the pellets were suspended again in PBS. The entire procedure was repeated for a total of three rounds. The three supernatants were then centrifuged at 20,000 × *g* for 15 min, and the three derivative pellets were pooled, split into two portions, which were in turn subjected independently to protein extraction as described below.

### DNA extraction and metagenome sequencing

DNA extraction from the MFM sample was undertaken using the QIAamp DNA Stool Mini Kit (Qiagen, Hilden, Germany), according to the manufacturer’s protocol.

The extracted DNA was quantified using the Qubit® 2.0 Fluorometer (Life Technologies, Carlsbad, CA, USA) and then subjected to next-generation sequencing. Libraries were generated according to the Nextera XT DNA Sample Preparation protocol (Illumina, San Diego, CA, USA). Normalized sample libraries were pooled and subjected to the cluster generation step using the cBOT cluster generation station, according to the Illumina TruSeq Paired-End Cluster Kit protocol. DNA sequencing was performed with the Illumina HiScanSQ sequencer, using the paired-end method and 93 bp of sequencing.

### Metagenome bioinformatics

Raw metagenomic data were demultiplexed using the Consensus Assessment of Sequence and Variation software (CASAVA, v.1.8.2) from Illumina, and reads passing the quality filters were submitted to the MG-RAST pipeline [40]. Briefly, the coding sequences were clustered at 90% of identity and taxonomically and functionally annotated. Taxonomic annotation was carried out according to the LCA approach, using the M5NR database [65] and the following filters: maximum e-value cutoff 10^−5^, minimum % identity cutoff 80%, and minimum alignment length cutoff 15. Functional annotation was achieved by performing a blastp search (v.2.2.29+) against the UniProtKB-Bacteria database (release 2013_11) with maximum e-value cutoff 10^−5^.

### Protein extraction

#### Individual microbial strains

Proteins were extracted from each individual microorganism according to two different methods: the first based on sample heating in SDS-based buffer and the second based on sample heating in SDS-based buffer plus bead-beating/freeze-thawing.

According to the first method, microbial pellets were resuspended in 100 μl of extraction buffer (2% SDS, 100 mM DTT, 20 mM Tris-HCl pH 8.8), incubated at 95°C for 20 min in agitation (500 rpm) in a Thermomixer Comfort (Eppendorf, Hamburg, Germany), and centrifuged at 20,000 × *g* for 10 min at 4°C, with the final supernatant being the protein extract.

According to the second method, microbial pellets were first resuspended in the above mentioned extraction buffer and incubated at 95°C as described above and then subjected to bead-beating combined with freeze-thawing as follows. A steel bead (5-mm diameter; Qiagen) was added to each sample, and samples were sequentially incubated at −80°C for 10 min, subjected to bead-beating for 10 min (30 cycles/s in a TissueLyser LT mechanical homogenizer, Qiagen), incubated at −80°C for 10 min and then at 95°C for 10 min, and subjected to a further 10-min bead-beating step. Samples were finally centrifuged at 20,000 × *g* for 10 min at 4°C, with the final supernatant being the protein extract.

Protein extracts obtained through both methods from each microorganism were quantified using the 2-D Quant Kit (GE Healthcare, Little Chalfont, UK), as per manufacturer’s instructions.

#### Mock microbial mixtures and fecal sample

Proteins were extracted from the 9MM, 4MUM, and MFM samples according to the method based on sample heating in SDS-based buffer plus bead-beating/freeze-thawing, as described above, and the volume of extraction buffer added was 500, 100, and 200 μl, respectively.

### Filter-aided sample preparation

Protein extracts obtained from 9MM, 4MUM, and MFM were subjected to on-filter reduction, alkylation, and trypsin digestion according to the FASP protocol [27], with slight modifications detailed elsewhere [66]. Briefly, protein extracts were diluted tenfold in 8 M urea, loaded into Microcon Ultracel YM-30 filtration devices (Millipore, Billerica, MA, USA), and centrifuged at 14,000 × *g* for 15 min. The concentrates were then diluted in 8 M urea and centrifuged again. After centrifugation, proteins were reduced in 10 mM DTT for 30 min and then alkylated in 50 mM iodoacetamide for 20 min. After five washes (three in 8 M urea and two in ammonium bicarbonate), trypsin solution was dispensed on the filter (1:100 enzyme-to-protein ratio), and the samples were incubated at 37°C overnight. Peptides were collected by centrifugation followed by an additional wash with an elution solution (70% acetonitrile plus 1% formic acid). Finally, the peptide mixture was brought to dryness and reconstituted in 0.2% formic acid to an approximate final concentration of 1 mg/ml.

Peptide mixture concentration was estimated by measuring absorbance at 280 nm with a NanoDrop 2000 spectrophotometer (Thermo Scientific, San Jose, CA, USA), using dilutions of the MassPREP *E. Coli* Digest Standard (Waters, Milford, MA, USA) to generate a calibration curve.

### LC-MS/MS analysis

LC-MS/MS analyses were carried out using an LTQ-Orbitrap Velos mass spectrometer (Thermo Scientific) interfaced with an UltiMate 3000 RSLCnano LC system (Thermo Scientific). After loading, peptide mixtures (4 μg per run) were concentrated and desalted on a trapping pre-column (Acclaim PepMap C18, 75 μm × 2 cm nanoViper, 3 μm, 100 Å, Thermo Scientific), using 0.2% formic acid at a flow rate of 5 μl/min. The peptide separation was performed at 35°C using a C18 column (Acclaim PepMap RSLC C18, 75 μm × 15 cm nanoViper, 2 μm, 100 Å, Thermo Scientific) at a flow rate of 300 nL/min, using a 485-min gradient from 1% to 50% eluent B (0.2% formic acid in 95% acetonitrile) in eluent A (0.2% formic acid in 5% acetonitrile).

The mass spectrometer was set up in a data-dependent MS/MS mode under direct control of the Xcalibur software (version 1.0.2.65 SP2), where a full-scan spectrum (from 300 to 1,700 m/z) was followed by MS/MS spectra. The instrument was operated in positive mode with a spray voltage of 1.2 kV and a capillary temperature of 275°C. Full scans and MS/MS spectra were performed in the Orbitrap with resolutions of 30,000 and 7,500 at 400 m/z, respectively. The automatic gain control was set to 1,000,000 ions, and the lock mass option was enabled on a protonated polydimethylcyclosiloxane background ion as internal recalibration for accurate mass measurements [67]. Peptide ions were selected as the ten most intense peaks of the previous scan, the signal threshold for triggering an MS/MS event was set to 500 counts, and dynamic exclusion was set to 30 s. Higher-energy collisional dissociation (HCD), performed at the far side of the C-trap, was used as the fragmentation method, by applying a 40% value for normalized collision energy, an isolation width of m/z 3.0, a Q-value of 0.25, and an activation time of 0.1 ms. Nitrogen was used as the collision gas.

### Metaproteome bioinformatics

#### Database construction

The protein database used for the metaproteomic analysis of the 9MM was generated based on previous genome sequencing data, which had been experimentally obtained from the nine individual microbial strains comprised within the 9MM [60]. The 4MUM protein database was generated as a subset of the 9MM database, restricted to the sequences belonging to the four individual microbial strains comprised within the 4MUM.

The protein database used for the metaproteomic analysis of the mouse fecal sample was instead constituted by a matched metagenome “core” (i.e., all the ORFs obtained from the metagenomic sequencing of the same sample; see the “Metagenome bioinformatics” section for details), to which additional reference proteome sequences retrieved from UniProtKB (release 2014_05) were appended, belonging to the following species: *Mus musculus* and *Glycine max* (soybean, being the main component of the mouse feed); fungal species belonging to the genera *Aspergillus*, *Candida*, *Cladosporium*, *Cryptococcus*, *Exophiala*, *Neocallimastix*, *Penicillium*, *Saccharomyces*, *Scleroderma*, and *Smittium*; and archaeal species belonging to the classes *Methanobacteria*, *Methanococci*, and *Methanomicrobia*. Supplementary information concerning the databases (number of sequences and average sequence length) are given in Additional file 8: Table S7.

#### Peptide identification

Peptide identification was performed using Proteome Discoverer (v.1.4; Thermo Scientific), with a workflow consisting of the following nodes (and respective parameters): Spectrum Selector for spectra pre-processing (precursor mass range: 350–5,000 Da; S/N Threshold: 1.5), Sequest-HT as the search engine (protein database: see above; enzyme: trypsin; maximum missed cleavage sites: 2; peptide length range: 5–50 amino acids; maximum delta Cn: 0.05; precursor mass tolerance: 10 ppm; fragment mass tolerance: 0.02 Da; static modification: cysteine carbamidomethylation; dynamic modification: methionine oxidation), and Percolator for peptide validation (FDR <1% based on peptide q-value). Results were filtered in order to keep only rank 1 peptides, and protein grouping was allowed according to the maximum parsimony principle.

#### Metaproteomic data analysis

The number of transmembrane domains within protein sequences was predicted using the TMHMM Server (v.2.0, http://www.cbs.dtu.dk/services/TMHMM). The taxonomic classification of the peptide sequences retrieved from UniProtKB records was carried out by means of Unipept (v.2.2.3, http://unipept.ugent.be) [68]. The interactive Pathways Explorer (iPath v.2, http://pathways.embl.de) was used to map proteins into metabolic pathways [48]. The MG-RAST and Unipept output data were parsed using in-house scripts, and graphs were generated using Microsoft Excel and Venn Diagram Plotter (http://omics.pnl.gov/software/VennDiagramPlotter.php).

## Availability of supporting data

Mass spectrometry data are available in the PeptideAtlas repository at http://www.peptideatlas.org/PASS/PASS00355.

## Additional files

Additional file 1: Table S1. Characteristics of the microbial strains used in this study. Additional file 2: Table S2. Number of ORFs, peptides, and PSMs identified in each sample, replicate, and run. Additional file 3: Figure S1. Evaluation of run reproducibility in the mouse fecal sample: correlation of PSM values among runs (left) and representation of identification overlap (right) at the ORF and peptide level. Additional file 4: Table S3. Microbial abundance distribution according to LCA analysis. Percentage abundance is based on PSM values. Additional file 5: Table S4. List of microbial protein families identified, along with the corresponding LCA taxonomic information. Additional file 6: Table S5. Taxonomic assignment of TonB-dependent receptor family members identified, according to the LCA approach. Additional file 7: Table S6. Taxonomic assignments concerning protein members of the Wood-Ljungdahl and the dissimilatory sulfate reduction pathways. Additional file 8: Table S7. Characteristics of the protein databases used in this work.

## Abbreviations

1-DE: one-dimensional electrophoresis
4MUM: four-microorganism unbalanced mixture
9MM: nine-microorganism mixture
ATP: adenosine-triphosphate
CFU: colony-forming unit
CoA: coenzyme A
DTT: dithiothreitol
FASP: filter-aided sample preparation
FDR: false discovery rate
LC: liquid chromatography
LCA: lowest common ancestor
MFM: murine fecal microbiome
MS/MS: tandem mass spectrometry
MS: mass spectrometry
PBS: phosphate-buffered saline
PSM: peptide-spectrum match
SRB: sulfate-reducing bacteria
TBDRF: TonB-dependent receptor family
TMD: transmembrane domain
