# Supplementary figures and images for: A straightforward and efficient analytical pipeline for metaproteome characterization
Source: Microbiome. 2014 Dec 10;2:49. doi: 10.1186/s40168-014-0049-2 (PMC4266899; doi:10.1186/s40168-014-0049-2)

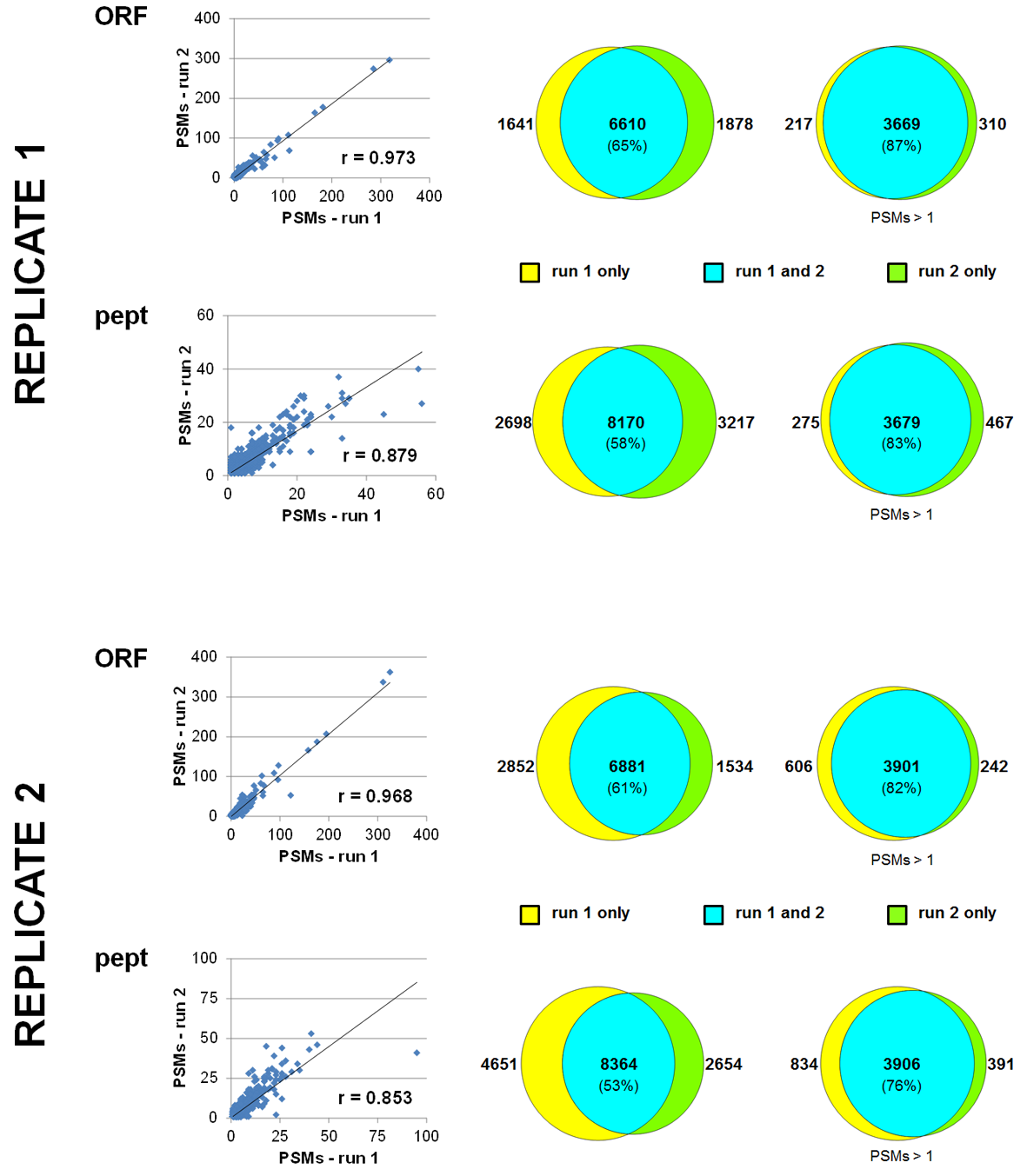

Supplement: Additional file 3: Figure S1. — Evaluation of run reproducibility in the mouse fecal sample: correlation of PSM values among runs (left) and representation of identification overlap (right) at the ORF and peptide level. [file 40168_2014_49_MOESM3_ESM.tiff]
